# Depression and Physical Activity Affect Diet Quality of Foreign-born Latina Women Living on the U.S.-Mexico Border

**DOI:** 10.3390/nu11061254

**Published:** 2019-06-02

**Authors:** Vanessa L. Errisuriz, Laura Delfausse, Alice P. Villatoro, Marisol D. McDaniel, Laura Esparza, Deborah Parra-Medina

**Affiliations:** Latino Research Institute, University of Texas at Austin, Austin, TX 78712, USA; ldelfausse@austin.utexas.edu (L.D.); avillato@austin.utexas.edu (A.P.V.); marisol.d.mcdaniel@austin.utexas.edu (M.D.M.); laura.esparza@austin.utexas.edu (L.E.); parramedina@austin.utexas.edu (D.P.-M.)

**Keywords:** diet quality, physical activity, depression, Latina women, immigrants

## Abstract

There is increasing evidence that depression may affect diet. However, little is known about the association between depression and diet quality among foreign-born Latinas. We hypothesized that depressive symptoms would be associated with poorer diet quality in foreign-born Latinas. Furthermore, we believed that physical activity (PA) would have a protective effect on diet quality for individuals experiencing depressive symptoms. Our study evaluated the diet (Healthy Eating Index) and PA (Actigraph GT3X activity monitors) of 534 foreign-born Latinas with and without depressive symptoms (Center for Epidemiological Studies Depression Scale). A series of logistic regression models were estimated to examine our hypotheses. As predicted, Latinas who were depressed had significantly lower odds of having a high-quality diet than non-depressed Latinas. Unexpectedly, among Latinas who met PA guidelines, depressed Latinas had a significantly lower probability of having higher-quality diets than their non-depressed counterparts. Our findings support current research stating that depressive symptoms are associated with lower Healthy Eating Index scores. More research is necessary to elucidate the relationship between PA and dietary quality of depressed Latinas. Innovative approaches to address mental health and the stressors that can compound its severity are needed to improve diet quality among foreign-born Latina women.

## 1. Introduction

Eating a healthy diet supports the maintenance of physical health, reducing the risk for chronic illnesses such as obesity, cardiovascular disease, type 2 diabetes, and several types of cancers. The Dietary Guidelines for Americans (2015–2020) recommend a healthy eating pattern that meets nutrient needs through the intake of vegetables, fruits, whole grains, and a variety of protein sources, low-fat or non-fat dairy products, and limited consumption of sugar, sodium, and solid fats [1]. Findings from nationally representative surveys indicate that Mexican-born Latinos living in the United States (U.S.) consume healthier diets (i.e., more fruits and vegetables, less sugar and fat) than their U.S.-born counterparts [2,3]. Unfortunately, diet quality becomes poorer the longer Latinos remain in the U.S. [4]. Latinos adopt unhealthy dietary behaviors as they adapt to life in the U.S., consuming more foods that are high in fat, sugar, and refined carbohydrates. It is not surprising, then, that much of the literature examining diet among foreign-born Latina women focuses on the association between acculturation and diet quality.

There are a variety of factors, besides acculturation, that may influence diet quality that are understudied in this population. For instance, higher levels of stress have been associated with greater preference for sweet foods; greater intake of energy-dense, nutritionally poor foods such as salty snack foods and caffeinated beverages; and decreased consumption of fruits, vegetables, meat, and fish [5,6,7]. Latinas who have emigrated to the U.S. face a variety of social and economic stressors, including challenges related to migration, resettlement, finances, and adapting to U.S. norms, all of which contribute to poorer mental health outcomes, such as depression [8]. However, little is known about the impact of depression on diet quality among foreign-born Latina women. This is concerning, as data from the National Health and Nutrition Examination Survey (NHANES) indicate that prevalence of depression among Latina women (10.5%) is higher than the national prevalence (8.1%) [9]. There is also evidence to suggest that the prevalence of depression is higher among foreign-born Latina women living in Colonias (i.e., unincorporated, impoverished, underserved Latino settlements) along the U.S.-Mexico border, with 20% reporting they had been told they had depression by a health care professional (i.e., physician or nurse) [10].

Research examining the association between depression and diet has primarily focused on understanding how the consumption of specific micro- and macronutrients (e.g., vitamin B-6, omega-3 fatty acids) or food groups (e.g., fruits and vegetables) contribute to the development of depression [11,12,13,14]. For instance, previous research has identified omega-3 fatty acids, [15] dietary tryptophan, and vitamin B-6, which are found in foods such as salmon, eggs, dairy, poultry, and whole grains, as important nutrients in the prevention of mood disorders [16]. Tryptophan and vitamin B-6, in particular, are instrumental in the production of serotonin, a neurotransmitter that regulates mood, emotion, wakefulness, sleep, and appetite. As such, higher intake of dietary tryptophan and vitamin B-6 are associated with higher levels of serotonin, which in turn, is related to fewer depressive symptoms [17,18]. 

Less is known about the potential influence of depression on diet quality. Findings from studies that have examined the influence of depression on diet quality are equivocal, with some indicating no impact from depression on dietary patterns and others indicating that depression negatively impacts dietary patterns [19,20,21]. There is a need to examine the influence of depression on dietary quality, which is more reflective of real-life scenarios, rather than on the intake of specific micro- or macronutrients as we do not consume foods and nutrients in isolation [22,23,24]. To our knowledge, there is non-existent to limited research examining the influence of depression on diet quality, especially among foreign-born Latina women [5,6,13,14,17]. 

Physical activity is an important correlate of both diet and depression. Among Latina women, in particular, self-reported regular engagement in physical activity is correlated with a healthier diet that contains servings of fruit and vegetables [25]. Engagement in higher levels of physical activity is also associated with decreased risk of depression and improved depression symptoms among individuals with depression [26,27]. Moreover, engagement in ≥10-minute bouts of moderate-to-vigorous physical activity is associated with fewer depressive symptoms among Latina women [28]. As such, physical activity may significantly impact the relationship between depression and diet.

Given the dearth of research on depression and diet quality among foreign-born Latina women, the current study examines: (1) the association between depressive symptoms and dietary quality, and (2) whether physical activity moderates this relationship in a cross-sectional sample of foreign-born Latina women living in Colonias along the U.S.-Mexico border. We hypothesized that depressive symptoms would be associated with poorer diet quality, consistent with previous research among different populations. We further hypothesized that engagement in physical activity would have a protective effect for individuals experiencing depressive symptoms such that those engaging in greater physical activity would have better quality diets than those engaging in less physical activity.

## 2. Materials and Methods

### 2.1. Study Design 

Data for this study were from Enlace, a promotora-led physical activity (PA) intervention implemented in the Lower Rio Grande Valley (LRGV) in South Texas between 2012–2016. The intervention was designed to implement a comprehensive, multilevel, community-based approach to promote moderate-to-vigorous physical activity (MVPA) among a particularly underserved and physically inactive segment of Latina women. Eight community resource centers (CRCs) in four predominantly Latino counties in the LRGV were recruited and randomly assigned to receive either the Enlace PA Intervention (four CRCs) or an attention-control condition (four CRCs). Promotoras were responsible for recruiting Latinas aged 18–64 years from Colonias in the CRC service areas. The study was conducted in three cohorts of approximately 207 women per cohort. Participants received the program (PA intervention or attention-control) if it was randomly assigned to the CRC from which they were recruited. The program for both conditions consisted of 16 group sessions followed by 24 weeks of maintenance. Participants completed self-reported questionnaires, and standardized measurement of participant anthropometrics and physical activity were conducted at baseline, immediately following the intervention (16 weeks), and immediately following maintenance (40 weeks). For the current study, we only used baseline data that was collected before program implementation. All study protocols were approved by the University of Texas Health Science Center San Antonio’s institutional review board. The study is registered in clinicaltrials.gov (NCT02046343). 

### 2.2. Participants

For the Enlace study, 620 Latina women living in Colonias on the U.S.-Mexico border were recruited. The majority of participants were foreign-born (86.1%). As such, the current study is focused on the foreign-born Latina women subsample (n = 534). On average, participants were 40.7 years old, spent approximately 17.6 years in the U.S., and had an average income of $1027 per month. 

### 2.3. Diet Quality 

*Healthy Eating Index*. This study assesses diet quality as a reflection of how well an individual’s diet conforms to dietary recommendations [16]. The Healthy Eating Index (HEI) is a useful index of overall dietary behavior, calculated from the Block Food Frequency Questionnaire [29,30], which has been validated for use among Latino populations [31]. Since the study began in 2012, we used the latest tool at the time, the HEI 2010. The HEI 2010 assesses diet quality as specified by the 2010 dietary guidelines. The HEI 2010 comprises twelve components, with varying maximum scores that contribute to the overall HEI 2010 score of 100: total fruit (max: 5), whole fruit (max: 5), total vegetables (max: 5), greens and beans (max: 5), whole grains (max: 10), dairy (max: 10), total protein foods (max: 5), seafood and plant proteins (max: 5), fatty acids (max: 10), refined grains (max: 10), sodium (max: 10), and energy from solid fats, alcohol, and added sugars (SoFAAS) (max: 20) [26]. Each HEI score was dichotomized based on median values due to non-normal distributions. Scores above the median reflect better dietary intake. Percentage of participants who met the cutoff for higher quality diet was 50.1% for total vegetables, 62.3% for greens and beans, 49.9% for total fruit, 50.7% for whole fruit, 49.9% for whole grains, 49.9% for dairy, 77.3% for total protein foods, 52.8% seafood and plant proteins, 52.0% for fatty acids, 50.1% for sodium, 50.1% for refined grains, 49.7% for SoFAAS, and 50.1% for HEI 2010 total score.

### 2.4. Depression

The Center for Epidemiological Studies Depression Scale (CES-D) assesses depressive symptoms in the past seven days with ten items (e.g., “You had trouble keeping your mind on what you were doing”) [32]. Participants rated items on a scale from 0 (rarely or none of the time) to 3 (most or all of the time). Responses were summed and their total score was dichotomized: CES-D scores <10 identified participants as not currently experiencing depressive symptoms and CES-D scores ≥10 identified participants as having likely depressive symptoms. Of the 534 participants, 179 (33.5%) met criteria for likely having depressive symptoms. 

### 2.5. Moderator 

*Physical Activity*. Participants wore an Actigraph GT3X 16Mb activity monitor (Actigraph Corp; Pensacola, FL, USA) in a belt around their waist, positioned on their right hip, over seven consecutive days. Research staff initialized the devices to collect data at a frequency of 30 Hz. Raw accelerometer data were downloaded and integrated into 60 s epochs using ActiLife software version 6 (Pensacola, FL, USA). The intensity of PA was measured using cut points for adults established by Freedson et al. (1998) [33]. For this study, we utilized total minutes per week in moderate-to-vigorous physical activity (MVPA) at baseline. Total minutes per week in MVPA was dichotomized into meeting physical activity guidelines (i.e., engaging in 150 minutes of MVPA per week) or not meeting physical activity guidelines. 

### 2.6. Covariates 

We collected participant demographic information (i.e., age, monthly income in dollars, age at which they emigrated to the U.S., and education level) via self-report at baseline assessments. We calculated the length of time a participant had spent in the U.S. by subtracting the age at which they emigrated to the U.S. from their current age. Trained research staff measured participant height (to the nearest 0.1 cm) and weight (to the nearest 0.1 kg) with the portable Seca 213 Stadiometer height-rod (Chino, CA, USA) and the Tanita Body Composition Analyzer SC-331S (Arlington Heights, IL, USA), respectively. Body mass index (BMI) was calculated as weight (kg)/height squared (m^2^).

### 2.7. Statistical Analyses

Analyses were conducted using the IBM Statistical Package for Social Sciences (SPSS version 25; Armonk, NY, USA). Examination of moderation effects was completed using Stata SE 15 (StataCorp; College Station, TX, USA) [34]. Due to the clustered nature of the data (i.e., individuals within CRCs), we used individual-level, fixed effects logistic regression models to examine the relationship between depressive symptoms and the binary diet quality outcome variables. We created dummy coded variables for each CRC to include in each model to account for this clustering (CRC 8 was the referent). Given that the present study investigates associations between individual-level variables and grouping/contextual effects are not of interest, utilizing fixed effects regression models was appropriate as they account for all variance associated at the cluster level [35].

First, we examined univariate statistics and bivariate associations using χ^2^ analyses and independent samples t-tests to examine the data and all study variables by depressive symptoms status (see Table 1). A series of logistic regression models were estimated to examine our study questions. For all outcomes, we first ran the main effects models, which included the fixed effects for the CRC, to examine the independent effect of depression on diet quality (Model 1). Next, fully adjusted models that included age, time spent in the U.S., BMI, income, education, cohort, and physical activity were estimated (Model 2). The final model tested the two-way interaction between depressive symptoms and physical activity on each dietary quality outcome (Model 3). To aid in the interpretation of statistically significant interaction terms from the logistic regression models, the predicted probabilities of having a higher-quality diet across depressive symptoms and physical activity were calculated and compared in post-estimation tests. Due to missing data ranging from 0.2% (depression) to 6.6% (met physical activity guidelines), only 462 participants were included in analyses.

## 3. Results

### 3.1. Sample Characteristics According to Depressive Symptom Status 

Participant characteristics did not significantly differ between participants with and without depressive symptoms (see Table 1). There was a trend for significance with age, where participants reporting depressive symptoms were slightly older than those who did not report depressive symptoms. HEI 2010 subcomponent scores did not significantly differ by depressive symptoms status; however, there was a strong trend toward significance for the total HEI 2010 score, such that participants with depressive symptoms had lower scores than those without depressive symptoms.

### 3.2. Are Depressive Symptoms Associated with Diet Quality among Foreign-Born Latina Women?

Table 2 and Table 3 present the results of the logistic regression models that examine the association of depressive symptoms on each of the dietary quality outcomes. Overall, considering the CRCs alone, Latinas who were depressed had significantly lower odds of having an overall high-quality diet than non-depressed Latinas (see Model 1.1 in Table 2). Similar patterns emerge when we further examine different aspects of their diet: controlling for the CRCs only, depressed symptoms were significantly associated with lower odds of having a high-quality diet with respect to total vegetable, total fruit, and SoFAAS (see Models 2.1, 3.1, and 6.1, respectively); this association trended toward significance for whole fruit (Model 4.1) and sea/plant protein (Model 5.1). Over and above the influence of the other covariates (i.e., age, cohort, education, income, time spent in the U.S., and BMI) and the moderator (i.e., meets physical activity guidelines), depressed Latinas were significantly less likely to have an overall high-quality diet than non-depressed Latinas (Model 1.2). This pattern was consistent for total vegetables, total fruit, whole fruit, sea/plant protein, and SoFAAS dietary components (see Models 2.2, 3.2, 4.2, 5.2, and 6.2). Depression was not associated with whole grain, total dairy, total protein, fatty acid, sodium, or refined grain HEI component scores (not shown in tables). In summary, the examination of the main effects models indicated that Latinas experiencing depression had poorer overall diet quality than Latinas without depression.

### 3.3. Does Physical Activity Moderate the Relationship between Depressive Symptoms and Diet Quality? 

**Figure 1:**
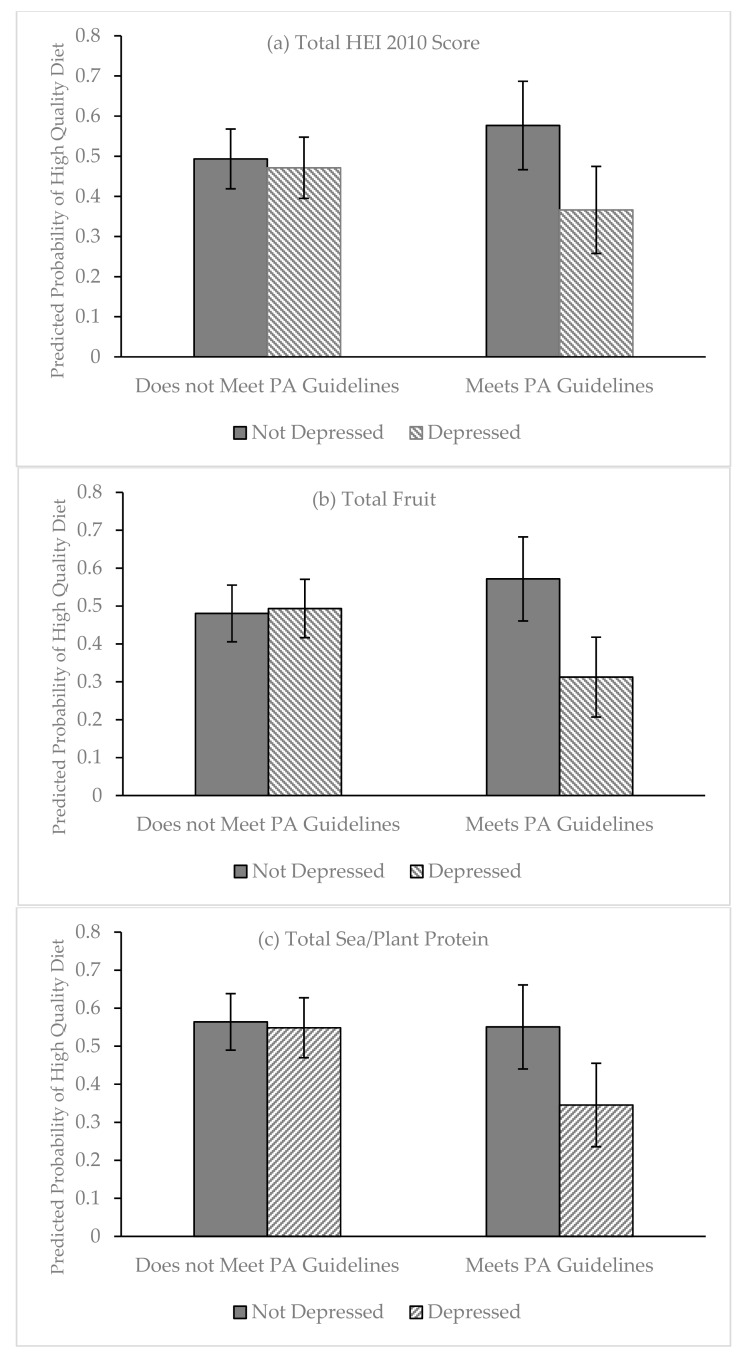
Predicted probabilities of high-quality diet across depressive symptoms and physical activity guidelines; Enlace (2012–2016; n = 462). Depressed Latinas, compared to non-depressed Latinas, showed significantly lower probabilities of having high-quality total HEI 2010 scores (*p* = 0.019), total fruit (*p* = 0.013), and total sea/plant protein (*p* = 0.029) when they met physical activity guidelines than when they did not.

To examine whether physical activity moderated the relationship between depressive symptoms and dietary quality, a two-way interaction between depression and physical activity was tested for each of the outcomes (see Table 2 and Table 3). This interaction was statistically significant for only two of the six outcomes: total HEI (Model 1.3) and total fruit (Model 3.3). There was a strong trend for significance for sea/plant protein scores (Model 5.3). Using the regression models for each of these outcomes, we estimated and compared the predicted probabilities of having a high-quality diet across depressive symptoms and physical activity (see Figure 1a–c). Overall, among foreign-born Latinas who did not meet physical activity guidelines, there were no statistically significant differences between depressed and non-depressed women in the probability of having high-quality total HEI (Figure 1a), total fruit (Figure 1b), and sea/plant protein (Figure 1c). Conversely, among women who met physical activity guidelines, depressed Latinas had significantly lower probabilities of having high-quality diets than their non-depressed counterparts. The difference-in-difference in high-quality diets between depressed and non-depressed Latinas and Latinas who met and did not meet physical activity guidelines was statistically significant for total fruit (*p* < 0.01) and approaching significance for total HEI (*p* = 0.051) and sea/plant protein (*p* = 0.052). In other words, the difference in the probability of having a high-quality diet between depressed Latinas and non-depressed Latinas was larger among Latinas who met physical activity guidelines compared to those who do not meet guidelines.

## 4. Discussion

Findings from the current study extend previous research by indicating that depressive symptoms are associated with diet quality among foreign-born Latina women. Our hypothesis that women experiencing depressive symptoms were more likely to have poor diet quality than those who do not report depressive symptoms was supported. Moreover, our results indicate that depressive symptoms may exert their influence on diet quality through specific dietary components of the HEI 2010, namely total vegetable, total fruit, whole fruit, sea/plant protein, and SoFAAS intake. These findings support current research that suggests individuals who are depressed are less likely to eat foods associated with higher quality diets, such as higher amounts of fruits and vegetables, than non-depressed individuals [13,23,24,25]. The current study further identified that the quality of specific components of diet, such as sea/plant protein and SoFAAS, might be particularly impacted among foreign-born Latinas experiencing depressive symptoms. These findings are consistent with previous research that indicates a high intake of processed foods and sugars is associated with an increased risk of depression [13,36]. 

The mechanisms through which depression may impact diet quality are not well understood. One study examining emotional eating behaviors in men and women with and without depression found that when exposed to stressors, those with depression were more likely to make poor dietary choices, choosing to eat energy-dense, snack-type foods. Furthermore, the association was only significant among women [37]. These findings suggest that maladaptive coping strategies in response to depression (e.g., emotion-oriented eating) are detrimental to diet quality, particularly among women. Sleep may also play a mediating role in the relationship between depression and diet quality. Sleep disturbances are a key feature of depression, and as such, they are included in most diagnostic criteria for depression [38]. There is increasing evidence that insufficient sleep (e.g. short sleep duration, poor sleep quality) is associated with increased food intake and poor diet quality [39]. Factors such as maladaptive coping and sleep disturbances may play a mediating role in the relationship between depression and diet. It is imperative to identify factors mediating the relationship between depression and diet quality to design effective interventions. 

However, the question remains: is a poor diet a consequence of depression, or does a poor diet cause depression? Findings from other research indicate that diet impacts the development of depression. A recent meta-analysis of prospective studies found that a healthy diet was associated with significantly lower risk of developing depressive symptoms [40]. Additionally, improving the diets of depressed individuals is associated with improved clinical outcomes, with the association strongest in females [41]. These findings suggest that depressed individuals may be more vulnerable to poor dietary patterns; however, they are also more likely to respond positively to dietary interventions. Therefore, improving the nutritional health of depressed women is likely a multi-directional issue that requires a multi-faceted treatment approach. 

While physical activity moderated the relationship between depressive symptoms and diet quality, it did not have the protective effect that we hypothesized: engagement in physical activity would ameliorate the negative impact of depressive symptoms on diet quality among our sample. Contrarily, among individuals with greater engagement in physical activity (i.e., meeting physical activity guidelines), those with depressive symptoms were more likely to have poorer diet quality and specifically, poorer total fruit, sea/plant protein, and total HEI 2010 scores. Depressive symptoms were not associated with diet quality among individuals not meeting physical activity guidelines. On the surface, this finding appears contradictory to previous work that demonstrates the positive effects of physical activity on depression. However, physical activity in the current study was assessed objectively with accelerometry. This limits the capability to determine the types of physical activity (e.g., occupational, transport, recreational) in which our sample of women were engaged. Previous work has indicated that the health benefits of physical activity may be limited to recreational physical activity [42]. It is posited that engagement in greater amounts of occupational and transport physical activity may not benefit health due to adverse working conditions for occupational and domestic physical activities (e.g., strenuous physical activities, long working hours), or risk factors related to commuting (e.g., exposure to air pollution, unfavorable climate) [42]. Of those in our sample who reported their occupation, women were primarily employed as service workers (e.g., hair stylist, waiter, caregiver, housekeeper), laborers (e.g., field worker, gardener), or sales clerks, occupations which are traditionally active. This suggests that the amount of activity our sample of women engage in throughout the day may be primarily due to occupation and/or transport, and is not recreational. Moreover, given the stressful nature of the occupations above, it is not surprising that meeting physical activity guidelines did not beneficially impact diet quality among women experiencing depressive symptoms. Future research should examine whether types of physical activity differentially impact the relationship between depressive symptoms and diet quality.

There are many factors other than mental well-being that can affect healthy eating, such as the food environment and food insecurity. About 78% of Latinos living in Colonias along the U.S.-Mexico border experience some food insecurity, putting them at an even higher risk for chronic disease because they are not able to access adequate and safe healthy foods [43]. They also rank access to health care as the second highest health concern in the community [10]. It is unsurprising, then, that Latinos with mental health issues are 50% less likely to seek medical care than non-Hispanic whites [44].

### Limitations

The cross-sectional nature of the study undermines our ability to determine whether depression influences diet quality or whether diet quality predicates the development of depression; however, it may also be that the relationship between depressive symptoms and diet quality is reciprocal. More longitudinal research is needed to elucidate the relationship between depression and diet among foreign-born Latina women. Additionally, the findings of the current study may not be generalizable to foreign-born Latina women residing in other parts of the U.S. or from other countries of origin (other than Mexico). However, this study provides a clear picture between well-being and diet quality among Mexican-origin women in South Texas that can influence future healthy lifestyle interventions in this specific population. Non-normal distributions necessitated the need to dichotomize diet quality variables, which can inflate the risk of type I error as well as underestimate the extent of variation in outcomes between groups [45]. For instance, individuals with scores that are close in range but on opposite sides of the cut point are characterized as being very different rather than very similar [46]. However, participant responses for diet quality variables were highly negatively skewed, with 50% or greater of respondents meeting recommendations for each HEI 2010 subcomponent score. Previous work examining diet quality among Mexican Americans residing in the U.S. has utilized dichotomous HEI 2010 scores due to non-normal distributions [47]. Finally, our measure of physical activity was not sensitive to the type of activity study participants engaged in (e.g., occupational, recreational, transport). Future research should assess whether types of activity have a differential impact on the relationship between depressive symptoms and diet quality among Latina immigrant women.

## 5. Conclusions

Depression is a significant public health issue among foreign-born Latina women [10]. The current study suggests that foreign-born Latina women experiencing depressive symptoms have lower odds of eating a high-quality diet than their non-depressed counterparts. Additionally, foreign-born Latina women who met physical activity guidelines and reported depressive symptoms had lower probabilities of eating high-quality diets than women who met physical activity guidelines and did not report depressive symptoms. This research has the potential to inform future healthy lifestyle interventions that promote physical activity and healthy eating. Public health researchers must integrate activities to foster mental well-being in healthy lifestyle interventions. Innovative approaches to address mental health, and the stressors that can compound its severity, are needed to improve diet quality among foreign-born Latina women.

## Figures and Tables

**Table 1:** Characteristics of Enlace Study sample by depressive symptom status.

Variable	Total Sample (n = 534) Mean (SD)	No Depressive Symptoms (n = 355) Mean (SD)	Depressive Symptoms (n = 179) Mean (SD)	*p*-Value
Age (years) ^a^	40.7 (9.9)	40.2 (9.7)	41.8 (10.1)	0.07
Time spent in the U.S. (years) ^a^	17.6 (9.8)	17.5 (9.9)	17.7 (9.8)	0.83
Income (monthly) ^a^	$1027.1 (831.8)	$1008.7 (810.4)	$1062.6 (877.3)	0.49
Body mass index (kg/m^2^) ^a^	31.3 (30.6)	31.1 (6.5)	31.7 (6.9)	0.38
	**n (%)**	**n (%)**	**n (%)**	
Education ^b^				
Less than high school	353 (66.1)	227 (63.9)	126 (70.8)	0.12
High school or more	181 (33.9)	128 (36.1)	52 (29.2)	
Cohort ^b^				
1	169 (31.6)	116 (32.7)	52 (29.3)	0.39
2	171 (32.1)	117 (33.0)	54 (30.3)	
3	194 (36.3)	122 (34.3)	72 (40.4)	
Met physical activity guidelines ^b^				
Yes	246 (49.3)	165 (49.3)	80 (49.1)	0.53
No	253 (50.7)	170 (50.7)	83 (50.9)	
	**Median (IQR)**	**Median (IQR)**	**Median (IQR)**	
HEI 2010 ^a^				
Total score	65.46 (13.1)	66.0 (14.1)	64.4 (11.5)	0.06
Total vegetables	4.1 (1.8)	4.4 (1.8)	4.0 (1.9)	0.11
Greens and beans	5.0 (1.1)	5.0 (1.0)	5.0 (1.2)	0.73
Total fruit	3.1 (3.1)	3.4 (3.1)	2.7 (3.0)	0.11
Whole fruit	3.8 (2.8)	4.2 (2.7)	3.6 (2.8)	0.17
Whole grains	2.7 (2.7)	2.8 (2.8)	2.6 (2.3)	0.11
Total dairy	3.8 (2.7)	3.9 (2.7)	3.5 (2.7)	0.18
Total protein	5.0 (0.0)	5.0 (0.0)	5.0 (0.0)	0.25
Total sea/plant protein	5.0 (1.6)	5.0 (1.5)	4.9 (1.6)	0.17
Refined grains	4.8 (4.7)	4.7 (4.7)	4.9 (4.9)	0.96
Fatty acid	10.0 (2.2)	10.0 (2.2)	10.0 (2.3)	0.80
Sodium	4.8 (3.4)	4.6 (3.5)	5.1 (3.5)	0.26
SoFAAS	16.9 (4.8)	17.1 (5.0)	16.2 (4.0)	0.14

^a^ denotes independent samples *t*-test; ^b^ denotes chi-square test for independence; SD = standard deviation; IQR = interquartile range.

**Table 2:** Logistic regression models associating higher total HEI 2010 scores and HEI total vegetable and fruit component scores, with depressive symptoms, physical activity, and two-way interaction between depression and physical activity; Enlace (2012–2016; n = 462).

	Total HEI 2010 Score OR (95% CI)			Total Vegetables OR (95% CI)			Total Fruit OR (95% CI)		
	**Model 1.1**	**Model 1.2**	**Model 1.3**	**Model 2.1**	**Model 2.2**	**Model 2.3**	**Model 3.1**	**Model 3.2**	**Model 3.3**
Depressive Symptoms (vs. no)	0.68 ^+^ (0.45–1.01)	0.62 ** (0.41–0.95)	0.93 (0.52–1.65)	0.57 ** (0.38–0.86)	0.56 ** (0.37–0.84)	0.70 (0.40–1.23)	0.64 * (0.43–0.95)	0.61 * (0.40–0.93)	1.06 (0.60–1.88)
Age (years)		1.02 ^+^ (0.99–1.05)	1.02 ^+^ (0.99–1.05)		1.00 (0.98–1.03)	1.00 (0.98–1.03)		1.01 (0.98–1.04)	1.01 (0.99–1.04)
Cohort 1 (ref)		-	-		-	-		-	-
Cohort 2		0.61 * (0.38–0.99)	0.61 * (0.38–0.99)		0.86 (0.54–1.38)	0.86 (0.54–1.38)		0.79 (0.49–1.27)	0.80 (0.49–1.28)
Cohort 3		1.05 (0.64–1.70)	1.08 (0.66–1.76)		0.93 (0.58–1.51)	0.95 (0.59–1.54)		1.42 (0.87–2.29)	1.48 (0.91–2.42)
Completed High School (vs. no)		0.91 (0.61–1.38)	0.91 (0.60–1.38)		1.14 (0.76–1.70)	1.14 (0.76–1.71)		0.94 (0.62–1.42)	0.94 (0.62–1.42)
Monthly Income (dollars)		0.72 (0.46–1.13)	0.70 (0.45–1.11)		0.70 (0.45–1.09)	0.69 (0.44–1.08)		0.51 ** (0.32–0.80)	0.49** (0.31–0.77)
Time in U.S. (years)		1.02 * (1.00–1.05)	1.02 * (1.00–1.05)		1.02 (0.99–1.04)	1.02 (0.99–1.04)		1.01 (0.99–1.04)	1.01 (0.99–1.04)
Body Mass Index (kg/m^2^)		1.01 (0.98–1.03)	1.01 (0.98–1.04)		1.01 (0.98–1.04)	1.01 (0.98–1.04)		0.99 (0.96–1.02)	0.99 (0.96–1.02)
Met PA Guidelines (vs. no)		1.12 (0.76–1.65)	1.46 (0.91–2.35)		0.86 (0.59–1.27)	1.01 (0.63–1.60)		1.00 (0.68–1.47)	1.45 (0.91–2.32)
Depressive Symptoms X Met PA Guidelines			0.43 * (0.19–1.00)			0.62 (0.27–1.42)			0.31 ** (0.13–0.72)

Note: All models adjust for the community resource centers (CRCs); estimates not included. OR = odds ratio; CI = confidence interval; * *p* ≤ 0.05; ** *p* ≤ 0.01; ^+^ *p* ≤ 0.10.

**Table 3:** Logistic regression models associating higher HEI whole fruit, sea/plant protein, and SoFAAS component scores, with depressive symptoms, physical activity, and two-way interaction between depression and physical activity; Enlace (2012–2016; n = 462).

	Whole Fruit OR (95% CI)			Sea/Plant Protein OR (95% CI)			SoFAAS OR (95% CI)		
	**Model 4.1**	**Model 4.2**	**Model 4.3**	**Model 5.1**	**Model 5.2**	**Model 5.3**	**Model 6.1**	**Model 6.2**	**Model 6.3**
Depressive Symptoms (vs. no)	0.70 ^+^ (0.47–1.04)	0.66 * (0.44–1.00)	0.72 (0.41–1.28)	0.68 ^+^ (0.42–1.02)	0.64 * (0.42–0.97)	0.93 (0.52–1.64)	0.63 * (0.42–0.95)	0.58 ** (0.38–0.88)	0.69 (0.39–1.23)
Age (years)		1.03 * (1.00–1.05)	1.03 * (1.00–1.05)		1.03 * (1.00–1.05)	1.03 * (1.01–1.06)		1.02 (0.99–1.04)	1.02 (0.99–1.04)
Cohort 1 (ref)		-	-		-	-		-	-
Cohort 2		0.81 (0.51–1.30)	0.81 (0.51–1.30)		0.66 ^+^ (0.41–1.06)	0.66 ^+^ (0.41–1.07)		0.62 * (0.39–1.00)	0.63 ^+^ (0.39–1.01)
Cohort 3		1.67 * (1.03–2.71)	1.68 * (1.04–2.73)		0.82 (0.51–1.33)	0.84 (0.52–1.37)		0.52 ** (0.32–0.84)	0.52 ** (0.32–0.86)
Completed High School (vs. no)		1.05 (0.70–1.58)	1.06 (0.70–1.59)		0.73 (0.49–1.10)	0.73 (0.48–1.10)		0.71 (0.47–1.07)	0.71 (0.47–1.07)
Monthly Income (dollars)		0.76 (0.49–1.19)	0.76 (0.48–1.18)		1.17 (0.75–1.84)	1.15 (0.74–1.18)		0.96 (0.61–1.50)	0.95 (0.60–1.49)
Time in U.S. (years)		1.00 (0.98–1.02)	1.00 (0.97–1.02)		0.99 (0.97–1.02)	0.99 (0.97–1.02)		1.02^+^ (0.99–1.04)	1.02^+^ (0.99–1.05)
Body Mass Index (kg/m^2^)		1.00 (0.98–1.03)	1.00 (0.98–1.03)		1.00 (0.97–1.03)	1.00 (0.97–1.03)		1.00 (0.97–1.03)	1.00 (0.97–1.03)
Met PA Guidelines (vs. no)		1.33 (0.91–1.95)	1.41 (0.89–2.25)		0.71 (0.49–1.05)	0.92 (0.58–1.47)		1.08 (0.74–1.59)	1.22 (0.76–1.94)
Depressive Symptoms X Met PA Guidelines			0.83 (0.36–1.91)			0.45 ^+^ (0.19–1.04)			0.69 (0.30–1.60)

Note: All models adjust for the community resource centers (CRCs); estimates not included. OR = odds ratio; CI = confidence interval; * *p* ≤ 0.05; ** *p* ≤ 0.01; ^+^ *p* ≤ 0.10.
